# Extended resections and other special cases in lung cancer surgery: Real‐world population‐based outcomes

**DOI:** 10.1111/1759-7714.13638

**Published:** 2020-09-01

**Authors:** Olli Helminen, Johanna Valo, Heidi Andersen, Johan Söderström, Eero Sihvo

**Affiliations:** ^1^ Department of Surgery Central Finland Central Hospital Jyväskylä Finland; ^2^ Department of Pulmonology Vaasa Central Hospital Vaasa Finland

**Keywords:** Extended indications, extended surgery, lung cancer, population‐based study, survival

## Abstract

**Background:**

Lung cancer invading outside a lobe centrally or peripherally, or presenting with synchronous or metachronous tumors, requires a special approach. Here, we aimed to evaluate the rate and outcomes of surgery of these patients in a medium‐volume practice using real‐world, population‐based data.

**Methods:**

All patients (*n* = 269) on whom lung cancer surgery was performed in Central Finland and Ostrobothnia between January 2013 and December 2019 were included. A total of 40 patients with sleeve (*n* = 18) or other extended resections (*n* = 9), multifocal diseases (*n* = 14), and other operated synchronous cancers (*n* = 3) required an extended or otherwise special surgical approach (extended group). Short‐ and long‐term outcomes were compared to high‐risk (*n* = 72) and normal patient groups (*n* = 157).

**Results:**

The rate of extended resection was 14.9%. The rates of PET‐CT (95%), invasive staging (35%), and brain imaging (42.5%) were highest in extended group compared to other groups. Extended group had larger and higher rate of stage III tumors than high‐risk and normal groups. All extended group patients underwent anatomic lung resection with better lymph node yield than the other two groups, with a neoadjuvant and/or adjuvant treatment rate of 70.0%. Major complications occurred in 7.5% in the extended group, 19.4% in the high‐risk group, and 6.4% in the normal group; at one year, alive and living at home rates were 88.2%, 83.3%, and 97.8%, and overall five‐year survival rates 75.6%, 62.4%, and 63.9% (*P* = 0.287), respectively.

**Conclusions:**

After guideline‐based evaluation, a significant rate of these special cases can be resected with a low complication rate and good long‐term survival in real‐world practice.

**Key points:**

**Significant findings of the study:**

- Extended resections for lung cancer include tumors spreading outside the lung
- The rate of extended resection was 14.9% in a population‐based setting
- Major complications occurred in 7.5% and five‐year survival was 75.6%

**What this study adds**

- Complication rate and long‐term outcome were similar compared to normal patients
- Guideline‐based evaluation results with excellent outcome in real‐world practice

## Graphical abstract

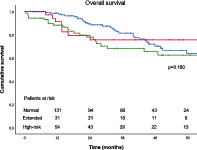

Extended resections for lung cancer include tumors spreading outside the lung. In this population‐based study, the rate of extended resection was 14.9% with major complications occurring in 7.5% and five‐year survival was 75.6%. Guideline‐based evaluation results with excellent outcome in real‐world practice.

## Introduction

Worldwide, the leading cause of cancer‐related mortality is lung cancer.^1^ For the modern treatment of this disease, several comprehensive guidelines exist.^1^, ^2^ In both the ESMO and ACCP guidelines, the preferred surgical resection for early stage lung cancer is anatomical resection, especially lobectomy.^1^, ^2^

Based on our previous population‐based study, the adherence to the modern guideline‐based approach had a major impact on outcomes.^3^ The best possible outcomes, on the other hand, in large STS and ESTS databases with 39% and 24% undergoing nonanatomic wedge resection, respectively, are most likely not reached.^4^ Therefore, regardless of any guidelines, variations in surgical practice of even early stage lung cancer exist.^4^

Sleeve lobectomy, rather than pneumonectomy, is recommended in centrally located tumors.^2^ Tumors invading the chest wall, spinal column, or other structures, or synchronous/metachronous cancers, if completely resectable, are special indications for surgery.^2^ For the treatment of these special cases, the importance of well‐established guidelines and book chapters is clear.^2^ Without adherence to these guidelines, many of these patients are likely to undergo nonsurgical therapy.^5^, ^6^

The reported rate of sleeve resections in large databases or in population‐based studies is only 1–2%.^4^, ^7^, ^8^ The outcome of these types of extended resections or the rate and outcomes of other special indications at the population level are unclear. In the present study, with the strict adherence of modern guidelines in a population‐based setting, we evaluated the rate and outcomes of lung cancer surgery in extended surgical indications and other special situations.

## Methods

### Design

In September 2012, a modern guideline‐based lung cancer surgery program was started at Central Finland Central Hospital by an experienced thoracic surgeon (ES).^3^ Lung cancer surgery in Ostrobothnia was centralized to Central Finland Central Hospital in October 2014. All patients diagnosed with primary lung cancer in Central Finland and Ostrobothnia between January 1, 2013, and December 31, 2019, were considered for inclusion in this population‐based cohort study. Follow‐up ended on 31 March 2020. Patients who underwent surgical resection were identified from hospital records and the prospective surgical database, and confirmed by data from the Finnish Cancer Registry (FCR). The acquisition of individual patient data from hospital records was approved by the local hospital districts. The National Institute for Health and Welfare of Finland (permissions no: THL/143/5.05.00/2015 and THL/1349/5.05.00/2015) and Statistics Finland (TK53‐1410‐15) approved the study.

### Data collection

All Finnish residents are listed by their individually unique and immutable 10‐digit national registration numbers in the hospital databases and several national databases. This allows for reliable identification of patients from hospital records and the Finnish Cancer Registry, as well as linkage of data. The Finnish Cancer Registry is population‐based and covers all parts of Finland. Registration includes the municipality and, therefore, the hospital district where the patient lives. According to Finnish health care policy, all hospital districts are responsible for arranging specialized care for their residents in its area. In Central Finland and Ostrobothnia, which had a total population of 456 976 as of 31 December 2017 (8.3% of the Finnish population), the treatment of lung cancer is organized by Central Finland Central Hospital and Vaasa Central Hospital, respectively. Using the histopathological, clinical follow‐up, and discharge registries of the two hospitals, all patients diagnosed with primary lung cancer who underwent lung resection with curative intent between 1 January 2013, and 31 December 2019, were identified. Cross‐linking of Finnish Cancer Registry data and hospital databases confirmed identification of all surgical cases. From 2015, a prospective surgical database established in Central Finland Central Hospital in 2012 provided the surgical cases. This database was not designed for study purposes and, therefore, the data for all study patients were collected from hospital records.

### Study groups and outcomes and definitions

The eighth edition of the TNM classification was used for staging. This required recoding all surgical patients accordingly. The study group (ie, extended group) included patients with extended central or peripheral local resection beyond the lung tissue, synchronous solid lung cancer in a different ipsilateral lobe or contralateral lung, metachronous lung cancer, or other low‐survival synchronous cancer (Table 1). Two patients undergoing sleeve segmentectomy, two patients with multifocal ground‐glass opacities, and six patients with synchronous breast cancer were included in the normal group. Outcomes were compared to a high‐risk group and normal‐risk nonextended group. High‐risk patients were defined by at least one of the following: age ≥ 80 years, FEV1 ≤ 50%, DLCO ≤50%, Charlson comorbidity index ≥5, maximal VO_2_ 10–15 mL/kg/minutes, or climbing only two flights of stairs (7.2 m). Complications were graded according to Clavien‐Dindo classification.^9^, ^10^

**Table 1:** Extended indications

	Number of patients
Extended resection	9
Chest wall	6
Pancoast	1
Chest wall and vertebral column	1
Pericardium	1
Sleeve resections	18
Bronchus Bronchus and pulmonary artery	12 3
Pulmonary artery	3
Multifocal disease	14
Ipsilateral separate lobe	5
Bilateral	5
Metachronous tumor	4
Synchronous malignancy	3
Esophageal cancer	1
Mesothelioma	1
Lung metastasis of bladder cancer	1

Overall number is 44 due to multiple indications in four patients.

The main outcomes of this study were differences in short‐term results (ie, hospital stay, complications, home discharge percentage, 90‐day mortality), ability to live at home one year after surgery, and long‐term and recurrence‐free survival. The ability to live at home was defined as the proportion of patients, out of all operated patients, living at home one year after surgery instead of a nursing facility, hospital, terminal care unit, or death. Only patients who completed one‐year follow up (operated before 31 March 2019) were included in the analysis regarding living at home one year after surgery.

### Statistical analysis

We constructed Kaplan‐Meier survival curves according to the life table method to visualize the crude all‐cause and recurrence‐free survival up to five years after surgery. Proportions, means, and median values of other measured variables were compared using the chi‐squared test, Mann‐Whitney U‐test, and Student's *t*‐test as appropriate. For patients who received neoadjuvant treatment, clinical stage was used instead of pathological stage. All statistical analyses were performed using IBM SPSS 25.0 (IBM corp., Armonk, NY, USA).

## Results

Over the seven‐year period, 269 patients underwent surgery for lung cancer in the Central Finland and Ostrobothnia hospital districts in Finland, with a mean age of 69.7 (SD 9.3) years (Table 2). Overall, the rate of video‐assisted thoracoscopic surgery (VATS) was 77.6% throughout the study period and 87.3% during the last four years.

**Table 2:** Baseline characteristics and preoperative patient evaluation between study groups

	All patients (*n* = 269)	Extended surgery (*n* = 40)	High‐risk (*n* = 72)	Normal patients (*n* = 157)	*P*‐value between groups
Age years, mean (SD)	69.7 (9.3)	68.7 (9.8)	74.6 (8.5)	67.7 (8.8)	<0.001^†^ ^,^ ^§^
Male, *n* (%)	172 (63.9%)	24 (60%)	45 (62.5%)	103 (65.6%)	0.508
Charlson comorbidity index					<0.001^†^ ^,^ ^‡^ ^,^ ^§^
0	26.0%	30.0%	9.7%	32.5%	
1	27.5%	22.5%	27.8%	28.7%	
2	21.2%	15.0%	26.4%	20.4%	
>2	25.3%	32.5%	36.1%	18.4%	
CCI, mean (SD)	1.7 (1.7)	1.8 (1.9)	2.6 (2.1)	1.3 (1.2)	
Fev1%, mean (SD)	80.4 (19)	77.4 (16)	72.6 (22)	84.8 (17)	0.013^‡^ ^,^ ^§^
DLCO/va, mean (SD)	79.9 (19)	78.2 (19)	71.6 (21)	84.3 (17)	0.058^§^
Stair climbing mean (SD)/median (IQR)	3.6 (0.7) 4 (3–4)	3.8 (0.4) 4 (4–4)	3.2 (0.9) 3 (2–4)	3.8 (0.4) 4 (4–4)	<0.001^†^ ^,^ ^§^
Percentage of patients climbing <3 flights	8.6%	0%	30.4%	0%	<0.001^†^ ^,^ ^§^

^†^ Significance (*P* < 0.05) between extended surgery and high‐risk groups.

^‡^ Significance (*P* < 0.05) between extended surgery and normal group.

^§^ Significance (*P* < 0.05) between high‐risk and normal group.

### Patients with extended indications for surgery and other study groups

In 40 patients, treatment required extended surgical resection outside the lung (*n* = 9, most often chest wall), sleeve resection (*n* = 18), treatment of multifocal disease (*n* = 14), and synchronous other poor‐outcome cancer (*n* = 3), Table 1. All patients underwent preoperative pulmonary function tests and physical evaluation, including stair‐climbing (Table 2). Patients in the extended group had worse pulmonary functions and more comorbidities than normal patients. The high‐risk group had the worst preoperative surgical risk profile (Table 2). The rate of preoperative staging was highest in the extended group, with PET‐CT performed in 95% of patients, invasive staging in 35%, and brain imaging in 42.5% (Table 3).

**Table 3:** Tumor characteristics

	All patients (*n* = 269)	Extended surgery (*n* = 40)	High‐risk (*n* = 72)	Normal patients (*n* = 157)	*P*‐value between groups
Histology, *n* (%)					<0.001^‡^ ^,^ ^§^
Adenocarcinoma	161 (59.9%)	19 (47.5%)	32 (44.4%)	110 (70.1%)	
Squamous cell cancer	78 (29.0%)	18 (45.0%)	33 (45.8%)	27 (17.2%)	
Other	29 (10.8%)	3 (7.5%)	7 (9.7%)	19 (12.2%)	
Tumor size cm, median (IQR)	2.5 (1.5–4.1)	3.7 (1.9–5.3)	3.1 (1.6–4.3)	2.2 (1.5–3.5)	0.003^‡^ ^,^ ^§^
PET‐CT, *n* (%)	207 (77.0%)	38 (95.0%)	59 (81.9%)	110 (70.1%)	0.002^‡^
Invasive staging, *n* (%)	60 (22.3%)	14 (35.0%)	23 (31.9%)	23 (14.6%)	0.002^‡^ ^,^ ^§^
Brain imaging, *n* (%)	38 (14.1%)	17 (42.5%)	7 (9.7%)	14 (8.9%)	<0.001^†^ ^,^ ^‡^
Resection type					
Pneumonectomy	2 (0.7%)	0	1 (1.4%)	1 (0.6%)	0.570
Lobectomy	168 (62.5%)	26 (65.0%)	37 (51.4%)	105 (66.9%)	0.025^§^
Segmentectomy	90 (33.5%)	14 (35.0%)	28 (38.9%)	48 (30.6%)	0.214
Wedge	9 (3.3%)	0	6 (8.3%)	3 (1.9%)	0.020
Lymph node dissection					0.477
No mediastinal samples	26 (9.7%)	2 (5.0%)	8 (11.1%)	16 (10.2%)	
N2 sampling	20 (7.4%)	1 (2.5%)	7 (9.7%)	12 (7.6%)	
Systematic N2 dissection	223 (82.9%)	37 (92.5%)	57 (79.2%)	129 (82.2%)	
Lymph node yield, median (IQR)	11 (7–15)	14 (11–17)	10 (5–14)	11 (8–16)	0.004^†^ ^,^ ^§^
R0 resection, %	99.6%	100%	100	99.4%	0.294
Oncological therapy					
Neoadjuvant, *n* (%)	31 (11.5%)	15 (37.5%)	7 (9.7%)	9 (5.7%)	<0.001^†^ ^,^ ^‡^
Adjuvant, *n* (%)	59 (21.9%)	21 (52.5%)	10 (13.9%)	28 (17.8%)	<0.001^†^ ^,^ ^‡^
Either neo‐ or adjuvant, *n* (%)	79 (29.4%)	28 (70.0%)	17 (23.6%)	34 (21.7%)	<0.001^†^ ^,^ ^‡^
Pathological stage, *n* (%)^2^					0.004^†^ ^,^ ^‡^
Carcinoma in situ	4 (1.5%)	1 (2.5%)	1 (1.4%)	2 (1.3%)	
IA1	17 (6.3%)	‐	4 (5.6%)	13 (8.3%)	
IA2	75 (27.9%)	8 (20.0%)	18 (25.0%)	49 (31.2%)	
IA3	41 (15.2%)	4 (10.0%)	10 (13.9%)	27 (17.2%)	
IB	28 (10.4%)	1 (2.5%)	12 (16.7%)	15 (9.6%)	
IIA	21 (7.8%)	2 (5.0%)	6 (8.3%)	13 (8.3%)	
IIB	43 (16.0%)	11 (27.5%)	14 (19.4%)	18 (11.5%)	
IIIA	31 (11.5%)	8 (20.0%)	5 (6.9%)	18 (11.5%)	
IIIB	7 (2.6%)	4 (10.0%)	1 (1.4%)	2 (1.3%)	
IIIC	1 (0.4%)	1 (2.5%)	‐	‐	
IVA	‐	‐	‐	‐	
IVB	1 (0.4%)	‐	1 (1.4%)	‐	

^†^ Significance (*P* < 0.05) between extended surgery and high‐risk groups.

^‡^ Significance (*P* < 0.05) between extended surgery and normal group.

^§^ Significance (*P* < 0.05) between high‐risk and normal group.

In the extended group, neoadjuvant therapy was given to 37.5%, and neoadjuvant and/or adjuvant treatment to 70.0% (Table 3). These rates were lower in the high‐risk (9.7% and 23.6%) and normal (5.7% and 21.7%) groups (*P* < 0.001). Rates of adjuvant therapy after neoadjuvant therapy and surgery were 42.5%, 33.3% and 0%, respectively. In stage IIA or higher tumors, the rate of either neoadjuvant and/or adjuvant therapy was in the extended group 88.5%. This rate was higher compared to the rate of 56.9% (*P* = 0.004) in the normal group or the rate of 51.9% (*P* = 0.004) in the high‐risk group.

### Quality of surgery

In the extended group, tumors were larger (median 3.7 [IQR 1.9–5.3]) and of higher stage (≥stage III in 32.5%) than in the high‐risk (≥stage III in 9.7%) and normal groups (≥stage III in 12.8%, *P* = 0.004). The rate of N2 disease was 15%, 10.2% and 6.9%, respectively, without statistical significance. In N2 patients of the extended group, the nodal burden with a median of one positive node and a ratio of 0.07 was lower than those of in the normal group with a median of two (*P* = 0.020) and a ratio of 0.19 (*P* = 0.007) or in the high‐risk group with a median of three (*P* = 0.035) and a ratio of 0.25 (*P* = 0.002). In the extended group, all patients underwent anatomic lung resection, and nearly all patients (92.5%) underwent systematic lymph node dissection, with better lymph node yield than the other two groups (Tables 3 and 4). The proportion of R0 surgery in the extended, high‐risk, and normal groups was 100%, 100%, and 99.4%, respectively.

**Table 4:** Patient outcomes

	All patients (*n* = 269)	Extended surgery (*n* = 40)	High‐risk (*n* = 72)	Normal patients (*n* = 157)	*P*‐value between groups
VATS, *n* (%)	209 (77.7%)	14 (35.0%)	63 (87.5%)	132 (84.1%)	<0.001^†^ ^,^ ^‡^
VATS during last four years, *n* (%)	145 (87.3%)	11 (45.8%)	40 (95.2%)	94 (94%)	<0.001^†^ ^,^ ^‡^
Anatomic lung resection	260 (96.7%)	40 (100%)	66 (91.7%)	154 (98.1%)	0.007^§^
Hospital stay, median (IQR)	5 (3–7)	5 (5–10)	5 (4–8)	4 (3–6)	<0.001^‡^ ^,^ ^§^
ICU stay, median (IQR)	0 (0–0)	0 (0–1)	0 (0–0)	0 (0–0)	<0.001^†^ ^,^ ^‡^ ^,^ ^§^
Discharged to					
Home, *n* (%)	222 (82.5%)	31 (77.5%)	53 (73.6%)	138 (87.9%)	0.020^§^
Complications, *n* (%)					
Any type	90 (33.5%)	13 (32.5%)	35 (48.6%)	42 (26.8%)	0.005^§^
Minor (grade I–II)	63 (23.4%)	10 (25.0%)	21 (29.2%)	32 (20.4%)	0.335
Major (grade IIIa–V)	27 (10.0%)	3 (7.5%)	14 (19.4%)	10 (6.4%)	0.008^§^
Living at home one‐year after surgery	212/229 92.6%	30/34 88.2%	50/60 83.3%	132/135 97.8%	0.001^‡^ ^,^ ^§^
Survival					
90‐day	98.5%	100%	94.4%	100%	0.004^§^
One‐year	94.8%	91.3%	88.3%	98.6%	0.003^§^
Five‐year	65.4%	75.6%	62.4%	63.9%	0.287

^†^ Significance (*P* < 0.05) between extended surgery and high‐risk groups.

^‡^ Significance (*P* < 0.05) between extended surgery and normal group.

^§^ Significance (*P* < 0.05) between high‐risk and normal group.

### Short‐term outcomes

In the whole population‐based cohort, the overall and major complication rates were 33.5% and 10%, including 1.5% 90‐day mortality (Table 4). These rates in the extended group were 32.5%, 7.5% and 0%, respectively. The high‐risk group had a significantly higher rate of overall complications (48.6% vs. 26.8%; *P* = 0.005), major complications (19.4% vs. 6.4%; *P* = 0.008), and 90‐day mortality (5.6% vs. 0%; *P* = 0.004) than the normal group. In the extended and high‐risk groups, hospital and ICU stays were significantly longer than in the normal group, though the median ICU stay was 0 in all groups. ICU stay ≥1 day was required for 32.5% of patients in the extended group, 11.1% in the high‐risk group, and 4.5% in the normal group.

### Long‐term outcomes

The rate of being alive and able to live at home one year after surgery in the whole population‐based cohort was 92.6%. After extended resection, this rate was 88.2% (Table 4). Compared to this rate and the rate in the high‐risk group (83.3%), the rate in the normal group was significantly higher (97.8%, *P* = 0.001).

**Figure 1:**
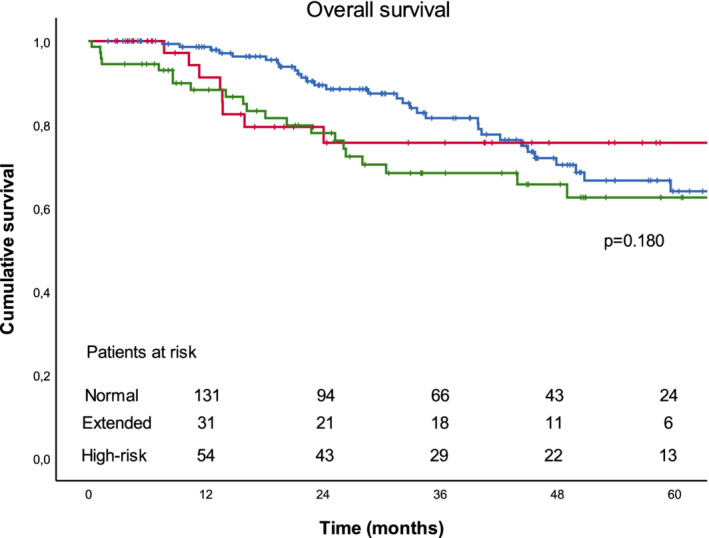
Overall survival stratified by study group.

**Figure 2:**
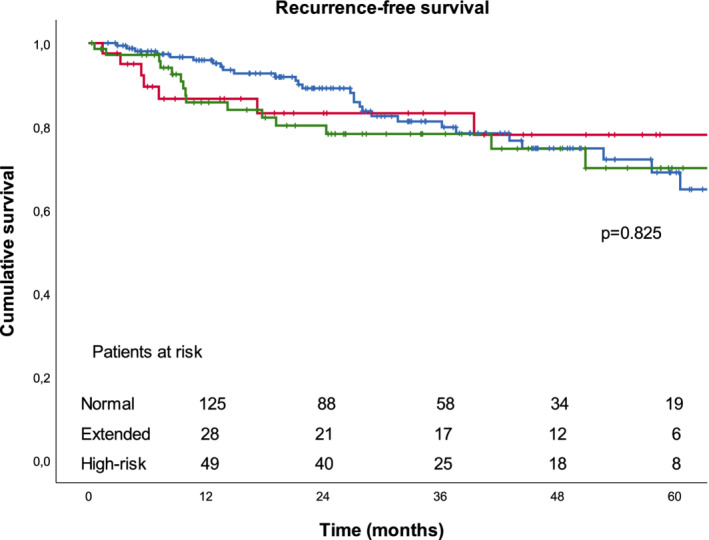
Recurrence‐free survival stratified by study group.

Overall survival at 90 days, one year, and five years after surgery for lung cancer at the population level was 98.5%, 94.8%, and 65.4%. Recurrence‐free survival at five years was 70.8%. Survival rates stratified by study group are presented in Table 4 and Figures 1 and 2. We found no significant difference in overall survival and recurrence‐free survival between the study groups.

## Discussion

Based on this real‐world experience, at the population level, patients undergoing extended resection or resections due to other special indications create a significant group comprising 15% of surgical lung cancer candidates. Surgery can be offered to these selected patients with excellent short‐ and long‐term outcomes. Complication rates were comparable to low‐risk patients undergoing mainly standard VATS‐lobectomy or segmentectomy. In this extended surgical group, the five‐year survival rate was 75.6%.

The population‐based design from two geographic areas in Finland is the major strength of this study. Unlike many other population‐based reports, we were not restricted to only registry‐based data, but had full access to complete patient data from hospital records. This resulted in more accurate collection of patient demographics, risk factors, and the exact surgeries performed. The available surgical reports are particularly important considering extended resection, which can be misleading if based only on surgical codes and registry data. Furthermore, complications based on hospital records provide accurate information without missing morbidity which often occurs in registry data. Complete follow‐up data were also available from multiple sources. However, the study had some weaknesses. The limited sample size, especially in the extended group, can cause confounding results by increasing the possibility of chance in our results. Limiting this possibility, however, is the fact that multiple factors, including complications, surgical quality, recurrence, and overall survival, were analyzed without discrepancies. Our study period included 2013–2019 with follow‐up extending to 31 March 2020. Actual five‐year survival was not available for all patients, therefore, survival rates were based on Kaplan‐Meier analysis.

The importance of well‐established guidelines and book chapters on the treatment of lung cancer in these special cases is clear.^2^ However, regardless of any guidelines, variations in surgical practice exist.^4^ In the case of multifocal and locally advanced tumors, patients are more likely to undergo nonsurgical therapy.^5^, ^6^ The rarity of various types of these special cases in our relatively small population is evident. The rate of 6.7% for sleeve resections is significantly higher than the 2.6% reported in a Danish population‐based series, most likely reflecting our very low pneumonectomy rate of 0.7%.^8^ The Danish rate of 4.4% in other extended resections is comparable to our rate of 3.4%.^8^ Although we had bronchovascular sleeve resections, a Pancoast tumor, a vertebral resection, and a resection after pneumonectomy, no potential resectable cancers infiltrating the superior vena cava, diaphragm, or aorta were staged during the study period. In this surgical series, the number of resected multifocal cancers (incidence 3.7%) is higher than that estimated in ACCP guidelines.^2^ By adding to these special cases, the incidence of other synchronous poor prognosis cancers (excluding six breast cancers) of 1.1% and metachronous lung cancers of 1.5%, a significant group of patients (15%) was formed for which we have to provide the best treatment in real‐world practice.

In this real‐world population‐based evaluation, the overall one‐ and five‐year survival after lung cancer surgery was 94.8% and 65.4%. These rates in the extended group were 91.3% and 75.6%, respectively. In the most recent 2018 Danish annual report, an overall one‐year survival of 91% and five‐year survival of 60% were detected at the population level in Denmark.^8^ In Finland between 2010 and 2014, one‐year and four‐year survival rates were 86.6% and 60.1%.^11^ Notably, even in the subgroup of high‐risk patients, the one‐ and five‐year survival rates (88.3% and 62.4%, respectively) were comparable to our national survival rates.^11^ A comparison of long‐term outcomes in the extended group is difficult due to miscellaneous patients. In a single center series after sleeve resection, the most common subgroup in the extended group, similar short‐ and long‐term outcomes as conventional lobectomy were reported, with five‐year survival of ~56%.^12^ After chest wall resection in the case of T3N0 disease, five‐year survival rates are consistently between 50% and 60%, and even up to 71%, with a published average of 55%, in Pancoast tumors.^2^ Survival after resection of multifocal (synchronous or metachronous lung cancer or other poor‐outcome malignancy) disease varies according to disease heterogeneity. For patient prognosis, the differentiation between multifocal lung cancer and intrapulmonary metastases is important. In bilateral tumors, survival rates in a retrospective single center series have reached 75%.^13^ A five‐year survival rate of 60% was detected in the SEER database analysis after surgery for a second primary lung cancer.^5^ Therefore, a five‐year survival rate of 75% at the population level in the extended group seems reachable.

A low mortality rate and low rate of major morbidity (0% 90‐day mortality and 7.5% major complication rate in this series) are prerequisites for advocating surgery over nonsurgical therapies in more extensive surgical cases. An additional argument for surgery is the 91.3% one‐year survival, which is superior compared to high‐risk patients in our study, and similar to Danish national outcomes.^8^ Furthermore, we were able to record the place of residence (home or other facility) for all patients one‐year postoperatively. With a rate of 88% of patients being alive and living at home one year after surgery, we were able to conclude that adequate performance status was maintained even after extensive resections. Previously, in the propensity matched comparison of patients undergoing chest wall resection or a more standard lung cancer resection, no differences were observed in long‐term quality of life.^14^ Overall, these kinds of short‐term outcomes justify surgery, as suggested in the guidelines, in various special cases requiring more extensive resections.

Both the overall short‐ and long‐term results at the population level in this study are equivalent or superior to previously published reports,^8^, ^11^ as were the outcomes in the extended group. The reason for these good outcomes could be a low resection rate. The actual rate in our districts is, however, significantly higher than the national surgical rate in Finland or any recently reported rate in Western countries.^3^, ^11^ Second, the surgical rates and outcomes in our area were improved by adherence to modern guideline‐based patient evaluation and treatment.^3^ In the extended groups, the PET‐CT rate of 95% and the brain imaging rate of 42.5% were the highest. Accurate staging has been reported to reduce the number of futile operations, and improve the quality of surgery and survival.^15^, ^16^ Standardized low‐technology exercise testing integrated in our routine risk assessment seemed to provide an excellent estimate of the physiological capacity of these patients.^3^ Third, the overall high rate of minimally invasive approaches, anatomic lung resections, R0 resections, and formal lymphadenectomies, together with a low rate of major morbidity and 90‐day mortality reflects the quality of the surgery. Overall number of examined lymph nodes in this population‐based series was 11 being significantly higher than that of seven in the SEER database.^17^ In the extended group, the 92.5% rate of formal mediastinal lymphadenectomies yielded the best median examined lymph node count of 14.^18^ The number of examined lymph nodes has been shown to be associated with improved survival.^18^ Fourth, in the extended group, the rate of neoadjuvant and adjuvant therapy was high and higher than those in the normal and high‐risk groups. Of patients with pathological stage IIA or higher cancers, 88.5% were given either neoadjuvant or adjuvant therapy. Despite clear recommendations, after neoadjuvant therapy and surgery, 42.5% of patients received further adjuvant therapy similar to esophageal cancer protocols.^19^ Finally, the rate of confirmed N2 nodal disease, an indicator of poor prognosis, was detected in only 15% of the extended group and was not statistically higher than those in other groups.^20^ Furthermore, the nodal disease burden of N2 patients, shown to correlate with survival, was lowest in the extended group with a median of only one positive node.^21^ Overall, the results indicate that after guideline‐based patient evaluation, complete radical resection combined with oncological therapies can be offered to these special cases, resulting in a low complication rate and good long‐term survival.

In conclusion, based on the current study, extended resection and resections due to other special indications create a significant group among surgical lung cancer patients (15%) at the population level. Despite more advanced tumors, or otherwise more complex clinical cases, surgery can be offered after thorough staging and risk‐assessment in real‐world practice, with excellent short‐and long‐term outcomes.

## Disclosure

The authors have no conflicts of interest or financial ties to disclose.
